# Research advances in the application of metabolomics in exercise science

**DOI:** 10.3389/fphys.2023.1332104

**Published:** 2024-01-15

**Authors:** Shuo Qi, Xun Li, Jinglun Yu, Lijun Yin

**Affiliations:** ^1^ School of Sport and Health, Shandong Sport University, Jinan, China; ^2^ School of Exercise and Health, Shanghai University of Sport, Shanghai, China; ^3^ School of Sport, Shenzhen University, Shenzhen, China

**Keywords:** metabolomics, exercise, metabolism, nutrition, biomarkers

## Abstract

Exercise training can lead to changes in the metabolic composition of an athlete’s blood, the magnitude of which depends largely on the intensity and duration of exercise. A variety of behavioral, biochemical, hormonal, and immunological biomarkers are commonly used to assess an athlete’s physical condition during exercise training. However, traditional invasive muscle biopsy testing methods are unable to comprehensively detect physiological differences and metabolic changes in the body. Metabolomics technology is a high-throughput, highly sensitive technique that provides a comprehensive assessment of changes in small molecule metabolites (molecular weight <1,500 Da) in the body. By measuring the overall metabolic characteristics of biological samples, we can study the changes of endogenous metabolites in an organism or cell at a certain moment in time, and investigate the interconnection and dynamic patterns between metabolites and physiological changes, thus further understanding the interactions between genes and the environment, and providing possibilities for biomarker discovery, precise training and nutritional programming of athletes. This paper summaries the progress of research on the application of exercise metabolomics in sports science, and looks forward to the future development of exercise metabolomics, with a view to providing new approaches and perspectives for improving human performance, promoting exercise against chronic diseases, and advancing sports science research.

## Introduction

**FIGURE 1:**
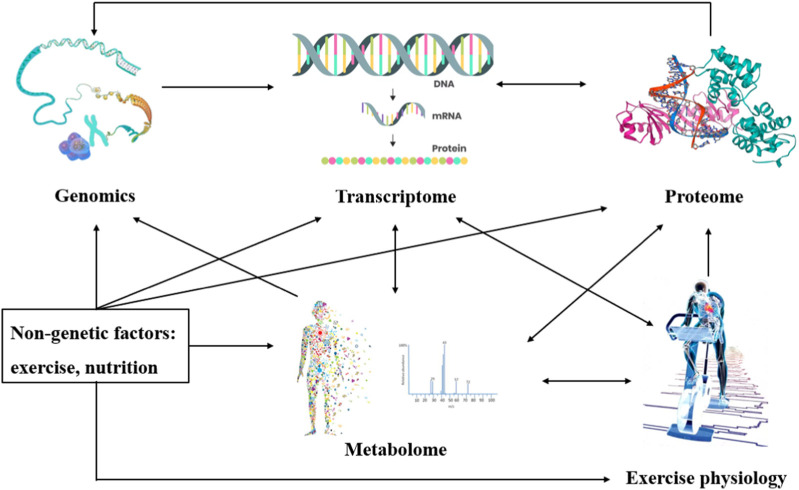
Interconnections between metabolomics and the environment. Metabolomics reflects the genome, transcriptome, and proteome and their interactions with the environment.

A biomarker is a biological characteristic that can be measured and evaluated in an organism or a biological sample, and can be used to indicate the physiological state of an organism, disease risk, disease progression or treatment effect, and other information (Qiu et al., 2023). Biomarkers can be molecules, cells, tissues, or physiological indicators, etc. Common biomarkers include genes, proteins, metabolites, hormones, and cell surface markers (Di Minno et al., 2022). Metabolomics is the quantitative analysis of small molecular weight metabolites (molecular weight <1,500 Da) in organisms, such as carbohydrates, amino acids, organic acids, nucleotides, and lipids, using mass spectrometry or magnetic resonance spectroscopy, and by studying the pattern of change of organisms or endogenous metabolites at a given moment (Kelly et al., 2020; Schranner et al., 2020; Belhaj et al., 2021; Shimada et al., 2021; Khoramipour et al., 2022). It is possible to investigate the interconnection and dynamic pattern of metabolites and physiological and pathological changes (Kelly et al., 2020; Kistner et al., 2021). Metabolomics reflects the genome, transcriptome, and proteome, as well as their interactions with the environment, and provides an ideal way to measure organismal phenotypes (Figure 1). Metabolomics data can provide useful insights into the biological effects of exercise, drug therapy, nutritional interventions, and more. Over the past decades, metabolomics has become a powerful tool for studying metabolic processes, identifying potential biomarkers, and deciphering metabolic reprogramming in various diseases to reveal the underlying mechanisms of relevant metabolic diseases (Belhaj et al., 2021).

Exercise can cause changes in the metabolism of many organs and tissues of the body, both acute and prolonged exercise, causing changes and adaptations in the body’s material metabolism and energy metabolism. Meanwhile, metabolites also regulate cellular signal release, energy transfer, and intercellular communication in the organism (Monnerat et al., 2020; Schranner et al., 2020; Belhaj et al., 2021; Klein et al., 2021; Martins Conde et al., 2021). However, exercise physiology has traditionally only been able to study a small number of genes, proteins, and metabolites and their responses or adaptations to exercise, with no more than 12 metabolites measured using traditional methods and only one to two metabolic pathways at a time, failing to comprehensively detect exercise-induced physiological changes in tissues or metabolic pathways (Belhaj et al., 2021; Khoramipour et al., 2022). The number of metabolites in the human body exceeds 110,000 compounds and the number of metabolic signaling pathways in the human body exceeds 40,000 (Wishart et al., 2020). In addition, invasive muscle biopsies are required to collect metabolic data in exercise physiology studies (Belhaj et al., 2021; Tokarz et al., 2021). While invasive muscle biopsies have successfully identified certain key metabolic pathways in the body, such as glycolysis and β-oxidation of free fatty acids, this invasive testing methodology limits the motivation of subjects to participate in the test and further limits the measurement of certain meaningful metabolic analyses, whereas the emergence of metabolomics has made it possible to conduct comprehensive, high-throughput, minimally or non-invasive metabolic studies in the field of exercise physiology (Castro et al., 2020). This review briefly introduces the exercise metabolomics technology and workflow, focuses on the research progress of exercise metabolomics applied in the field of sports science, and looks forward to the future development direction of exercise metabolomics. The technique provides researchers with an effective research tool, which helps to improve the practical ability and depth of theoretical understanding of sports performance and chronic disease exercise control.

## Introduction to exercise metabolomics

With the continuous development of histological techniques, exercise physiology is increasingly using metabolomics to probe organismal phenotypes, reveal metabolic pathways through the measurement of endogenous compounds, and identify biomarkers associated with exercise performance and fatigue, which has been termed “exercise metabolomics” (Kelly et al., 2020; Zhou et al., 2021). In 2007, Pohjanen et al. (2007) introduced exercise metabolomics to exercise science by performing 90 min of stationary cycling on 24 healthy men, collecting blood samples for gas chromatography-mass spectrometry (GC-MS) analysis, and identifying 420 metabolites, of which 34 were significantly altered, with an emphasis on the role of the most valuable biomarkers (glycerol and asparagine), which demonstrated, for the first time, the potential of non-targeted GC-MS metabolomics to provide a useful tool for the identification of metabolic pathways associated with exercise performance and fatigue. Metabolomics by mass spectrometry may provide a comprehensive and unbiased approach to studying the metabolic effects of exercise interventions (Pohjanen et al., 2007). Currently, the most commonly used biological samples in exercise metabolomics studies are blood and urine, and most studies use non-targeted metabolomics techniques, with mass spectrometry being the most commonly used detection and analysis platform in exercise metabolomics studies.

The main question in exercise science research is to understand how exercise induces physiological adaptations in the body, such as an increase in muscle strength or aerobic metabolic capacity, and how these adaptations affect health. But what are the molecular network mechanisms and metabolic pathways that govern how humans adapt to exercise and gain health benefits (Sakaguchi et al., 2019; Blackburn et al., 2020; Febvey-Combes et al., 2021; Babu et al., 2022)? These questions remain to be fully elucidated, and the study of exercise metabolomics will greatly enrich the understanding of these molecular network mechanisms and metabolic pathways.

### Workflow of exercise metabolomics studies

**FIGURE 2:**
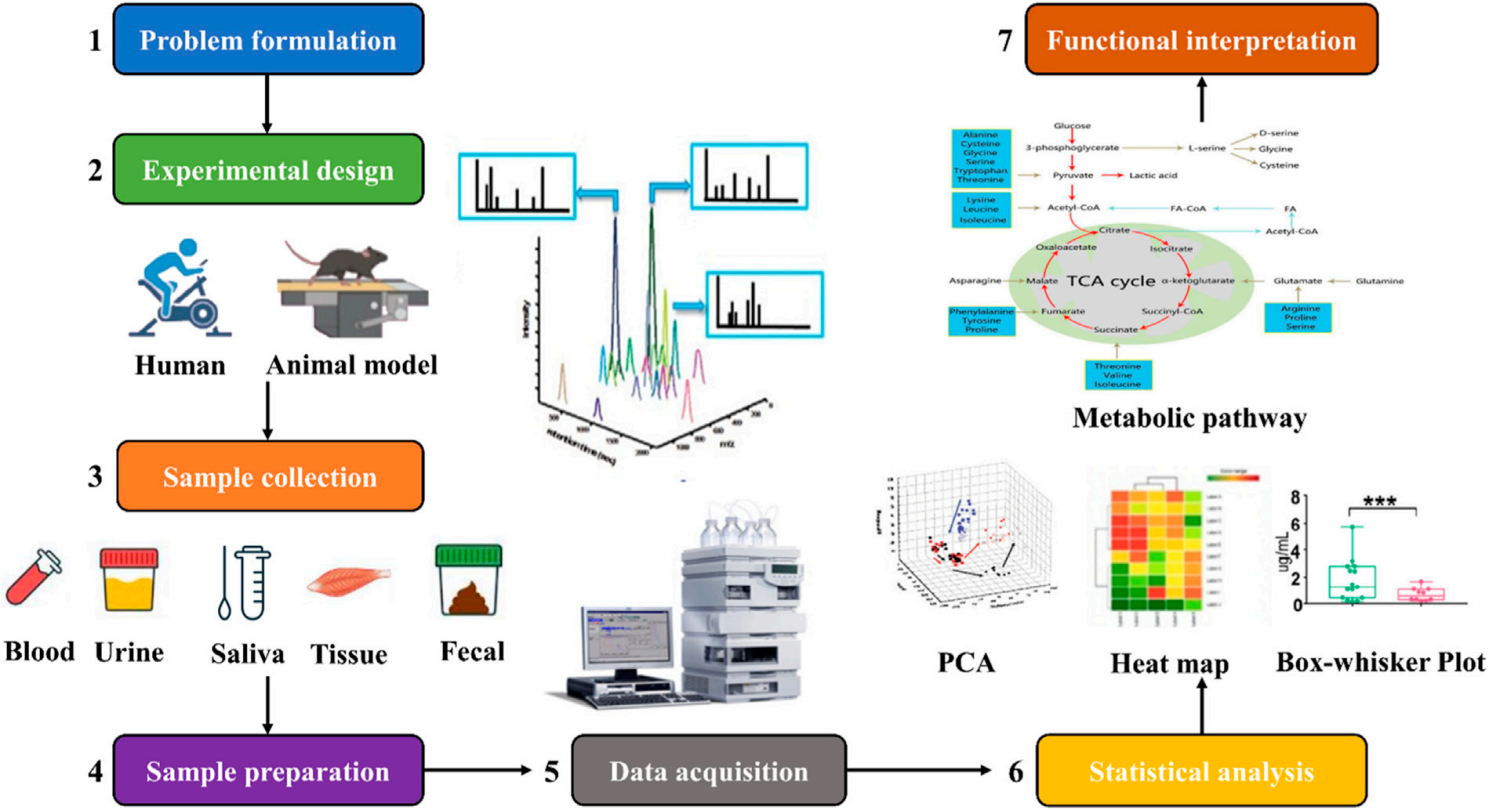
Workflow of exercise metabolomics studies. The workflow of exercise metabolomics research mainly includes problem formulation, study design, sample collection, sample preparation, data collection, statistical analysis, and functional interpretation.

As shown in Figure 2, the workflow of exercise metabolomics research includes 1) identifying the biological question of the study; 2) developing a study design based on the biological question of the study; 3) collecting experimental samples; 4) preparing the samples; 5) analyzing the samples and acquiring the data using one or more analytical platforms; 6) statistically analyzing compounds based on the biological question and experimental design to determine metabolic differences between different groups of samples; 7) researchers use software tools and databases to integrate the detected compounds with the biological context to further enable metabolic pathway enrichment analysis, metabolite mapping, and visualization, which can help inform future research questions and experimental designs (Belhaj et al., 2021; Khoramipour et al., 2022).

### Sample collection and sample preparation

In human metabolomics research, the most common sample types are blood, urine, saliva, sweat, and fecal samples, with muscle biopsies and other types of tissue biopsies accounting for a lower percentage, and endogenous metabolites in the samples better reflect the physiological changes in the organism (Kelly et al., 2020; Khoramipour et al., 2022). Sample type, sample quantity, and sample storage conditions are the keys to metabolomics experiments, each biological sample has advantages and disadvantages, the most commonly used types of biological samples in exercise metabolomics research are blood and urine, and the collection method is minimally invasive or non-invasive, and easy to be accepted by the subjects. Once collected, biological samples must be further processed or extracted to convert them into a state suitable for chemical analysis (Khoramipour et al., 2022).

## Chemical analysis platform and data analysis

Chemical analysis platforms used for sample characterization in metabolomics research include Gas Chromatography-Mass Spectrometry (GC-MS), Nuclear Magnetic Resonance (NMR), and Liquid Chromatography-Mass Spectrometry (LC-MS) (Table 1) (Kelly et al., 2020; Nicolaides et al., 2021; Khoramipour et al., 2022). The process of data processing and information analysis in metabolomics mainly includes the analysis of data, extraction of biological information, and functional interpretation of biological connotations (Khoramipour et al., 2022). The data generated in metabolomics studies have multivariate characteristics, when the number of metabolites in a given sample reaches hundreds or thousands, multivariate analysis methods capable of dealing with related variables are required to achieve reliable comparisons between multiple samples based on the whole set of variables. For example, Principal Components Analysis (PCA), Partial Least Squares-Discriminant Analysis (PLS-DA), and Orthogonal Partial Least Squares-Discriminant Analysis (OPLS-DA) (Castro et al., 2020; Khoramipour et al., 2022).

**TABLE 1:** Advantages and disadvantages of different chemical analysis methods.

Analytical platform	Advantages	Disadvantages
Nuclear magnetic resonance (NMR)	Fast Analysis	Low sensitivity
	High resolution technology	Small library of reference compounds
	No derivatisation required	More than one peak per metabolite
	Simple preparation	Limited to hydrophilic molecules
	Highly reproducible	Expensive instrumentation
	Low cost	
	Structure can be determined	
	Fully automatable	
	Non-destructive methods	
Gas chromatography–mass spectrometry (GC–MS)	High sensitivity	Lower yield
	High resolution	Requires chemical derivatisation
	Large linear absorption range	Not suitable for thermally unstable compounds
	Suitable for volatile compounds	High molecular weight
	Large library of commercial and public reference spectra	Only for compounds that can volatilise
	Highly reproducible	Complicated preparation process
	Mostly automatable	
Liquid chromatography–mass spectrometry (LC–MS)	Usually no derivatisation required	Low throughput
	Can be used with a variety of separation methods	Limited library of reference spectra
	Multiple samples can be analyzed simultaneously	Expensive instrumentation
	Suitable for a wide range of compounds (polar and non-polar)	Chromatographic separation required
	Most sensitive metabolomics technology	Advanced training required
	Good automation capabilities	Destructive methods

## Advances in exercise metabolomics research

There is a multifactorial dosage relationship between the effects of exercise on metabolic pathways, including the intensity of exercise, the duration of exercise, and the frequency of exercise (Hargreaves and Spriet, 2020; Osawa et al., 2021). These factors can strongly influence the metabolic changes in the organism after exercise. In turn, the type and program of exercise, the level of exercise, and even exercise nutrition can also affect the body’s metabolism. In addition, the effect of exercise on the metabolomics of chronic diseases is also a current research hotspot, which provides new perspectives on the prevention and treatment of chronic diseases.

### Effects of different exercise durations on body metabolism

There are some differences in the categories of metabolites induced in the body by different modes of exercise. For example, a short period of acute exercise can immediately cause changes in the metabolic pathways of skeletal muscle substrate utilization, and the changes in tricarboxylic acid (TCA) cycle metabolites are obvious after 1 h of exercise (Kelly et al., 2020; Tabone et al., 2021). Amino acids such as leucine, isoleucine, asparagine, methionine, lysine, glutamine, and alanine decreased significantly after 14 h of exercise, reflecting the large magnitude of changes in amino acid levels after acute exercise (Sakaguchi et al., 2019). Changes in plasma fatty acids, ketone bodies, bile acids, and triglycerides also showed changes that can last for several hours after acute exercise, eventually returning to pre-exercise levels (Sakaguchi et al., 2019). For example, weight lifting and dumbbell training, resistance exercises such as pull-ups. Sakaguchi et al. found that within 24 h of a short period of acute exercise in the body, Significant changes in metabolites such as carbohydrates, TCA circulating metabolites, fatty acids, carnitine, ketone bodies, amino acids, and their derivatives were found (Sakaguchi et al., 2019). Nayor et al. (2022) found that dimethylguanidinopentanoic acid and glutamate levels were reduced after a short period of acute exercise (Nayor et al., 2022). Therefore, short-duration acute exercise can cause more substantial changes in metabolites related to energy metabolism.

**FIGURE 3:**
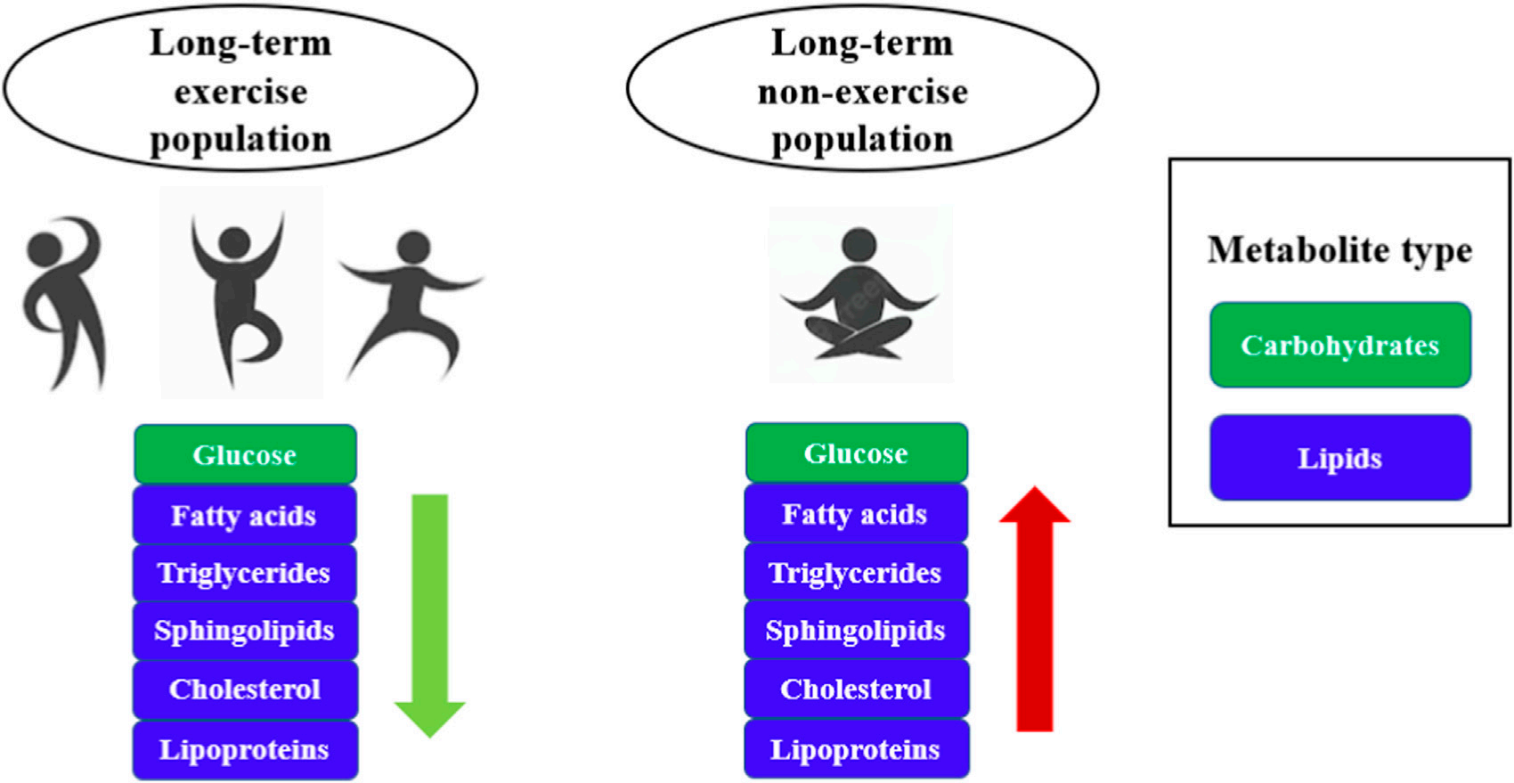
Comparison of metabolites between the long-term exercising population and the non-exercising population. The workflow of exercise metabolomics research mainly includes problem formulation, study design, sample collection, sample preparation, data collection, statistical analysis, and functional interpretation.

Kujala et al. (2013) compared amino acid levels in several groups of twins who had been exercising consistently for several decades and found that the fatty acid composition of the long-term exercising population gradually shifted from a saturated to an unsaturated state and that glucose and isoleucine levels were lower (Kujala et al., 2013). As shown in Figure 3, changes in metabolites such as glucose, fatty acids, and triglycerides were observed in both long-term exercising and long-term non-exercising populations, but higher levels of fatty acids, triglycerides and cholesterol existed in long-term non-exercising populations, which are prone to chronic metabolic diseases such as dyslipidemia, hypertension, cardiovascular disease, stroke, type 2 diabetes and metabolic syndrome (Mendham et al., 2021; Remie et al., 2021; McClain et al., 2022). However, people who exercise for a long period can accelerate the utilization of energy substances, reduce the accumulation of fat, and lower the levels of fatty acids, triglycerides, and cholesterol, which is conducive to the maintenance of healthy body weight as well as lowering the risk of chronic diseases (Table 2) (Tzimou et al., 2020; Bihlmeyer et al., 2021; Koay et al., 2021; Lemonakis et al., 2022). In the future, we will focus on the far-reaching effects of long-term exercise on weight loss and health.

**TABLE 2:** Effects of different exercise durations on key metabolites.

Duration of different exercises	Changes in key metabolites
Acute exercise of short duration	Leucine, isoleucine, asparagine, methionine, lysine, glutamic acid, glutamine, alanine, dimethylguanidinovaleric acid and other metabolites ↓
Long period of exercise	Glucose, isoleucine, fatty acids, triglycerides, cholesterol, sphingolipids, lipoproteins and other metabolites ↓

### Effects of different exercise intensities on body metabolism

**FIGURE 4:**
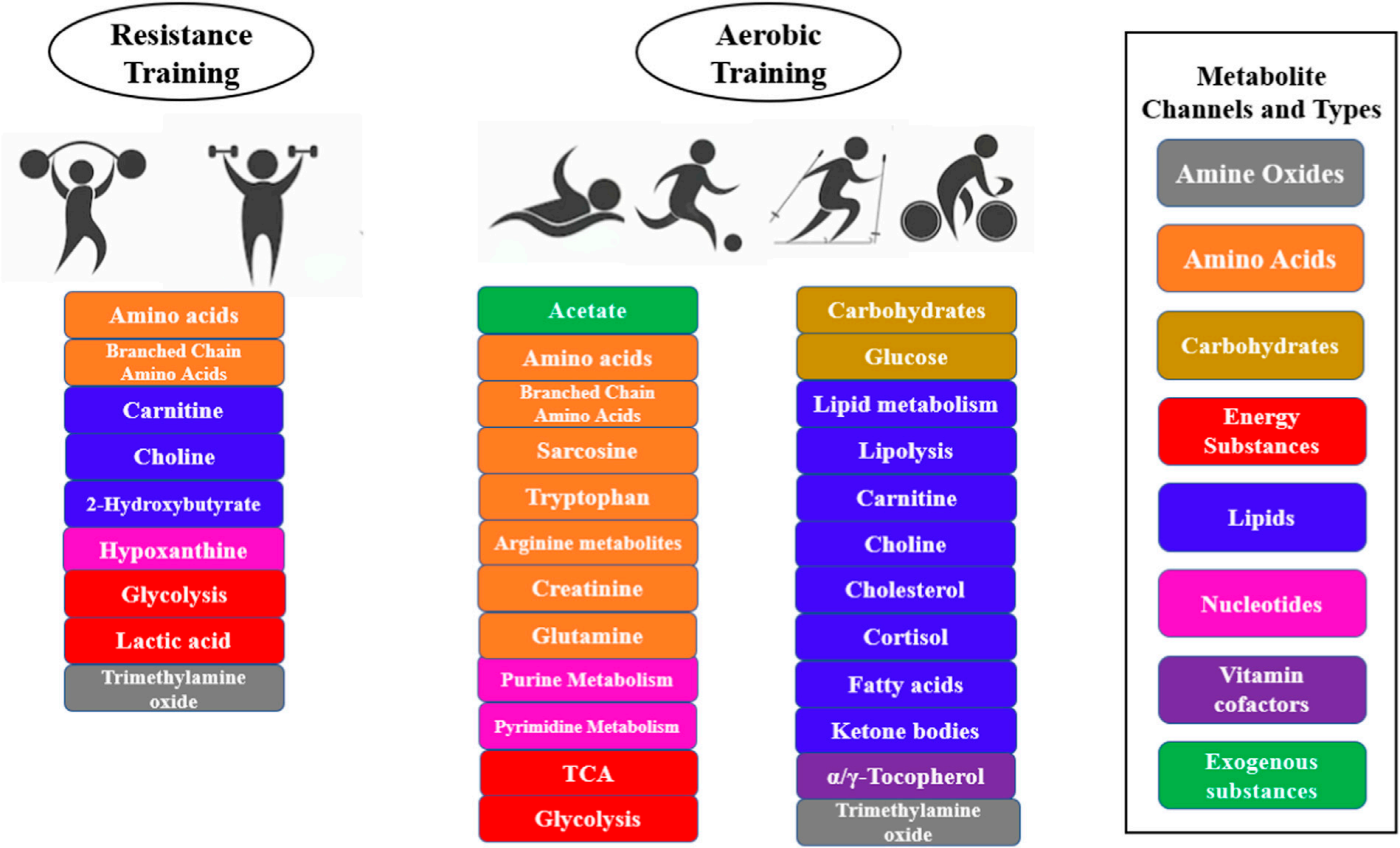
Metabolites, metabolic pathways of different exercise intensity category interventions.

The most common metabolic pathways induced by exercise in the body are changes in fatty acid metabolism, fat mobilization, lipolysis, TCA cycle, glycolysis, amino acid metabolism, carnitine metabolism, purine metabolism, and cholesterol metabolism (Kelly et al., 2020). Different exercise intensities have different effects on the body’s metabolism, with low-intensity aerobic training being dominated by aerobic metabolic pathways, with increases in the TCA cycle, fatty acid metabolism and amino acid metabolic pathways, and high-intensity resistance training being dominated by anaerobic metabolic pathways, with an increase in glycolysis and purine metabolism pathways (Figure 4). The effect of exercise intensity on metabolic profiles was also present in outstanding athletes, Al-Khelaifi et al. (2018) collected blood samples from outstanding athletes in different sports and analyzed the changes in 743 metabolites based on an LC-MS platform, and found that outstanding athletes with low-intensity endurance training had higher levels of serum sex hormones (testosterone and progesterone), and lower levels of diacylglycerol and eicosanoids; while high-intensity strength-trained elite athletes had higher levels of phospholipids and xanthines (Al-Khelaifi et al., 2018). Aerobic training mainly includes, running, cycling, football, and endurance sports such as swimming. Resistance training usually consists of high load, low repetition muscle contractions during a race (Granacher et al., 2016). Examples include weightlifting training, polymetric training, or machine-based training that includes upper and lower body exercises such as squats, jumps, weighted sprints, push-ups, and pull-ups (Fiorenza et al., 2019). This type of training is known to promote metabolic changes that facilitate anaerobic processes and increase muscle strength. Exercises such as gymnastics, martial arts and rock climbing also exhibit a high resistance component. In addition, endurance and resistance components are often combined, for example, in exercise interventions that combine running with weight training. Many sports also have significant endurance and resistance components, such as sprinting, boxing and rugby.

The type of sport also affects the body’s metabolic differences, such as marathons, track, boxing, cycling, football, rowing, rugby swimming, etc. There are differences in metabolic changes in different sports, and the reason for metabolic differences in different sports is mainly due to the different proportion of the energy supply system, such as weightlifters with the phosphate energy supply system, rowers with the glycolysis energy supply system, and marathon athletes with the aerobic oxidative energy supply system (Al-Khelaifi et al., 2018). Even the metabolism of athletes in different positions in the same sport varies, e.g., there are metabolic differences between football goalkeepers and field players participating in the same game, and these differences are likely to be caused by exercise intensity and exercise duration (Blackburn et al., 2020; Schader et al., 2020; Bester et al., 2021; Pugh et al., 2021). In addition, athletes in endurance sports have significantly increased levels of glycolytic products, TCA cycle intermediates, nucleotide metabolites, acylcarnitines, and branched-chain amino acids, which are frequently associated with aerobic metabolic pathways. Resistance training studies have shown significant increases in levels of creatine, choline, guanidine acetate, and hypoxanthine and decrease in creatinine levels in athletes in strength and explosive events, metabolites that are commonly associated with muscle growth, intracellular buffering, and methyl regulation (Khoramipour et al., 2022).

### Effects of different levels of exercise on the body’s metabolism

The level of exercise also affects the body’s metabolic differences, and Enea et al. used metabolomics for the first time to differentiate between metabolite changes in trained and untrained women, who underwent a 75% maximal oxygen uptake test, and then collected urine samples to analyze the metabolite changes based on an NMR platform, and found that the metabolites of creatine, lactic acid, pyruvic acid, alanine, β-hydroxybutyric acid, acetate, and hypoxanthine significant differences between groups (Enea et al., 2010).

Not only is there a difference in metabolism between trained and untrained individuals, but also the same athletes and different levels of exercise affect metabolism. Schader et al. (2020) found that slower marathon runners with lower levels of aerobic metabolism capacity had drastically altered levels of metabolites, with significant changes in phospholipids and amino acids (Schader et al., 2020). In contrast, the metabolomic alterations in good athletes were characterized by higher levels of phosphatidylcholine after the race (Høeg et al., 2020). San-Millan et al. (2020) found increased levels of circulating metabolites in TCA and elevated amplitude of lactate accumulation in good cyclists (San-Millán et al., 2020). In addition, Prado et al. collected urine samples from football players and analyzed their metabolic changes during competition based on an LC-MS platform, identifying 1,091 metabolites, of which 526 metabolites showed significant changes, including significant increases in the levels of glucose, uric acid urea, fatty acyls, carboxylic acids, steroids and steroid derivatives, and significant decreases in the levels of potassium (Khoramipour et al., 2022). Hudson et al. (2021) based on an NMR platform analyzed the metabolite changes in blood, urine, and saliva samples of outstanding rugby players, and found that the energy metabolism pathways of rugby, as a sport with high exercise intensity, mainly include glycolysis, TCA cycle, and gluconeogenesis (Hudson et al., 2021). Moreira et al. (2018) analyzed the urinary metabolites of outstanding swimmers and found that creatine, ketone bodies, phosphate, and nitrogen-containing compounds can be used as urinary metabolites to assess the outstanding swimmers’ exercise performance, which can be accurately assessed. Athletes’ performance, which can accurately assess their physiological status and provide a scientific basis for the development of athletes’ training load programs (Moreira et al., 2018).

## The effect of sports nutrition on the body’s metabolism

Kirwan et al. (2009) found that post-exercise intake of sugars and caffeine and analysis of metabolite changes in blood samples based on an NMR platform revealed a significant decrease in blood glucose levels, a significant increase in ketone body levels, and a significant increase in plasma levels of lactic acid and alanine (required for gluconeogenesis), which is the first study of sports nutrition metabolomics (Kirwan et al., 2009). Kozlowska et al. (2020) explored the effects of beetroot juice supplementation on the metabolism of fencing, and urine samples were collected to identify changes in metabolites based on an LC-MS platform, and significant changes in the metabolism of tyrosine, tryptophan, epinephrine, and norepinephrine were detected, which can help provide a scientific basis for the development of training load programs for athlete (Kozlowska et al., 2020). Yan et al. (2018) investigated the modulatory effects of ginseng supplementation on the metabolic patterns of professional athletes and explored the mechanism of ginseng’s antifatigue effects. Their metabolite analysis of blood samples from athletes based on a GC-MS platform revealed that American ginseng significantly modulated serum metabolism, significantly decreasing serum creatine kinase and blood urea nitrogen levels (Yan et al., 2018). Cronin et al. (2018) explored the effects of physical activity and protein intake on gut microbial composition and function, and genomics and metabolomics evaluations revealed significant changes in gut microbial composition and function with increased physical activity, with the gut virome significantly changing with increased physical activity in participants who received daily supplementation with whey protein (Cronin et al., 2018). Among participants receiving daily whey protein supplementation, the diversity of the gut virome changed significantly, suggesting that exercise and nutrition can significantly influence the composition and function of the gut microbiota. Therefore, metabolomics serves as an assessment tool to facilitate the design of personalized and fine-tuned exercise training and nutritional guidance programs for athletes, which can help to maximize athletic performance. In addition, Zhang et al. (2020) explored the effects of a 6-month exercise and dietary intervention on serum metabolites in men with insomnia symptoms, collecting blood samples from subjects for metabolite analysis based on a GC-MS platform, and found that the effects of exercise on sleep were mainly related to amino acid, carbohydrate and lipid metabolism, whereas the effects of diet on sleep were related to carbohydrate, lipid and organic acid metabolism (Zhang et al., 2020). Thus, metabolomics provides new insights into the effects of physical activity and diet on sleep quality.

### Exercise metabolomics in chronic disease prevention and treatment research

Contrepois et al. (2020) analyzed more than 600 metabolite changes in blood samples collected based on an LC-MS platform using a variety of histological approaches (targeted and untargeted metabolomics, lipidomics, proteomics, and transcriptomics), and showed that exercise has a significant effect on energy metabolism, oxidative stress, inflammation, tissue repair and its regulatory pathways in diabetic patients (Contrepois et al., 2020). Shi et al. (2019) found that Exercise can alter myocardial and skeletal muscle metabolism in heart failure model rats, and the metabolic pathways of taurine and hypotaurine metabolism and carnitine synthesis have a certain regulatory effect on alleviating heart failure, thus providing an effective target for the treatment of patients with heart failure (Shi et al., 2019). Siopi et al. (2019) studied the effect of exercise training with different intensities on male patients with metabolic syndrome and collected blood samples to analyze the changes of metabolites based on the LC-MS platform (Siopi et al., 2019). They found that resistance training induced the strongest metabolic changes, and the metabolites of branched-chain amino acids, alanine, carnitine, choline, and betaine had larger changes, indicating that exercise has beneficial effects on important serum biomarkers in patients with metabolic syndrome, which can help optimize the exercise guidelines for the people with risk of metabolic syndrome and improve the exercise prescription (Siopi et al., 2019). Palmnas et al. (2018) analyzed the blood samples of obese people based on the NMR platform and found that the obese people had the best metabolite changes. Blood samples and found that serine and glycine concentrations were lower in the obese population, which can help to find molecular targets for the treatment of chronic metabolic diseases in obese populations (Palmnäs et al., 2018). Liu et al. (2021) collected blood samples from children with metabolic syndrome and analyzed the changes in metabolites based on the LC-MS platform, and found that exercise combined with dietary interventions induced 59 metabolites (glycine, serine, and threonine metabolisms, nitrogen metabolism, TCA cycling, and phenylalanine, tyrosine, and tryptophan biosynthesis, etc.) to changes, thus providing early diagnostic biomarkers for the treatment of metabolic diseases such as obesity (Liu et al., 2021).

### Analysis of metabolic pathways by exercise

Exercise training induces changes in the body’s metabolic pathways such as lipid metabolism, TCA cycle, glycolysis, amino acid metabolism, carnitine metabolism, and purine metabolism (Kelly et al., 2020). Positive effects on cardiovascular health and mitochondrial biogenesis exist in populations that engage in chronic low-intensity aerobic training, where energy is produced through oxidative phosphorylation (Rivera-Brown and Frontera, 2012). Therefore, the activation of aerobic metabolic pathways and the increase in the TCA cycle, fatty acid β-oxidation metabolism and amino acid metabolism pathways, which allows for the presence of lower levels of fatty acids, triglycerides, and cholesterol in the organism, can accelerate the utilisation of energy substances and reduce the accumulation of fat, which is conducive to the maintenance of a healthy body weight as well as reducing the risk of chronic diseases, in addition to increasing the variety of energy substances burned during exercise (Pellegrino et al., 2022). Chronic metabolic adaptations in prolonged exercise populations typically affect metabolic pathways such as glycolysis, protein synthesis, amino acid consumption, and nucleotides.

In addition to improving muscle strength and metabolic health with short bursts of high-intensity resistance training, it also induces muscle hypertrophy. Resistance training is associated with metabolic changes that contribute to improved anaerobic capacity, muscle health, and glycolytic metabolism (Krzysztofik et al., 2019). Resistance training is mainly dominated by anaerobic metabolic pathways, with an increase in glycolytic and purine metabolic pathways. One of the most significant metabolic adaptations induced by resistance training is the increase in protein synthesis and depletion of amino acids, which are necessary to increase muscle mass (Shen et al., 2021; Gehlert et al., 2022). In addition, the pathways involved in nucleotide synthesis—the production of RNA, DNA and phospholipids required for cellular membranes—are activated (Pellegrino et al., 2022). There is an increase in the rate of ATP hydrolysis and the rate of nucleotide turnover, an increase in the accumulation of lactic acid in metabolites following acute resistance training and an increase in the ability to promote glycolytic metabolic adaptations (Gehlert et al., 2022). Metabolic adaptations in elite athletes are characterised by increased fuel substrate utilisation, fatty acid β-oxidation, oxidative stress, steroid biosynthesis and protein anabolic pathways (Cai et al., 2022).

Physical activity has many benefits for both physical and mental health, as studied through metabolomic analysis of metabolites released from tissues such as skeletal muscle, bone and liver. These metabolites can influence the body’s metabolic adaptations and improve cardiovascular health, reduce inflammation and increase muscle mass. Aerobic training increases mitochondrial content and oxidative enzymes, while resistance training increases muscle fibres and glycolytic enzymes. Acute exercise leads to changes in amino acid metabolism, lipid metabolism and cellular energy metabolism as well as cofactor metabolism and vitamin metabolism. Chronic exercise leads to changes in amino acid metabolism, lipid metabolism and nucleotide metabolism and improves lipid metabolism, thereby improving cardiovascular risk factors and skeletal muscle adaptations. The study of exercise-induced metabolites is a growing field with the potential to reveal more metabolic mechanisms and tailor exercise programs for optimal health and exercise performance.

### Potential limitations of exercise metabolomics

Many early exercise metabolomics studies lacked statistical rigor, e.g., extensive use of multivariate statistics, small sample sizes, and a single platform for metabolic analyses (Khoramipour et al., 2022). Furthermore, there were deficiencies in the sensitivity and specificity of the biomarkers used in most exercise metabolomics studies (Schranner et al., 2021). With the continuous advancement of histological technologies, metabolomics data alone may not be sufficient to fully characterize complex physiological changes. There is still potential for further improvements in the study design of many exercise metabolomics studies. In particular, exercise-related parameters or measurements, such as exercise intensity and exercise duration, have a strong influence on metabolic changes after exercise training. Researchers should incorporate and quantify these parameters more consistently in study designs, which would facilitate comparisons between studies. Another important goal of exercise metabolomics research is to routinely use and integrate more histological (proteomics, genomics, transcriptomics) techniques in study design. Metabolomics should not be an “island,” and the integration of multi-omics data will help researchers to further understand the interactions between genes, proteins, metabolites, and the environment, and to gain a deeper understanding of the effects of exercise on the organism.

### Summary and outlook

Exercise metabolomics provides researchers in the field of exercise science with an effective research tool to search for potential biomarkers and therapeutic targets by detecting metabolite changes in a variety of biological fluids and tissues after exercise in athletes and patients with chronic diseases, thus helping to improve the practical ability and depth of theoretical understanding of exercise performance and exercise prevention and treatment of chronic diseases.

With the continuous maturation of the technology and the deepening of the research, future exercise metabolomics research will further evaluate the exercise performance of outstanding athletes, so that their physiological conditions can be accurately assessed, which will provide a scientific basis for the development of precise training load programs for athletes and help coaches to cultivate outstanding athletes in a more effective way. In addition, mass spectrometry-based metabolomics testing is important for the treatment of metabolic disorders and provides clinicians with effective targets for the treatment of metabolic disorders, which is helpful for the treatment of chronic metabolic disorders. Meanwhile, mass spectrometry-based metabolomics studies will cover more subjects and will identify more metabolites. It is expected that more points of interest will emerge in the field of exercise metabolism and sports nutrition, focusing on the use of metabolomics findings to further design personalized and precise nutritional regimens to maximize the health benefits of physical performance and exercise. Finally, from the category of research groups, exercise metabolomics should focus more on human studies and more on practical orientated, reality-based experimental designs, collecting non-invasive sample collection methods based on urine and saliva. Such a trend will make exercise metabolomics research more informative and popular with participants, and lead to better and more accessible research tools for researchers, athletes, and coaches.
